# A Novel Equipment-Free Paper-Based Fluorometric Method for the Analytical Determination of Quinine in Soft Drink Samples

**DOI:** 10.3390/s23115153

**Published:** 2023-05-28

**Authors:** Vasiliki C. Tsaftari, Maria Tarara, Paraskevas D. Tzanavaras, George Z. Tsogas

**Affiliations:** Laboratory of Analytical Chemistry, School of Chemistry, Faculty of Sciences, Aristotle University of Thessaloniki, GR-54124 Thessaloniki, Greece

**Keywords:** paper-based analytical devicues, fluorometric determination, quinine, UV irradiation, simple imaging devices, soft drink samples

## Abstract

A simple, equipment-free, direct fluorometric method, employing paper-based analytical devices (PADs) as sensors, for the selective determination of quinine (QN) is described herein. The suggested analytical method exploits the fluorescence emission of QN without any chemical reaction after the appropriate pH adjustment with nitric acid, at room temperature, on the surface of a paper device with the application of a UV lamp at 365 nm. The devices crafted had a low cost and were manufactured with chromatographic paper and wax barriers, and the analytical protocol followed was extremely easy for the analyst and required no laboratory instrumentation. According to the methodology, the user must place the sample on the detection area of the paper and read with a smartphone the fluorescence emitted by the QN molecules. Many chemical parameters were optimized, and a study of interfering ions present in soft drink samples was carried out. Additionally, the chemical stability of these paper devices was considered in various maintenance conditions with good results. The detection limit calculated as 3.3 S/N was 3.6 mg L^−1^, and the precision of the method was satisfactory, being from 3.1% (intra-day) to 8.8% (inter-day). Soft drink samples were successfully analyzed and compared with a fluorescence method.

## 1. Introduction

Quinine (QN) is a quinidine alkaloid, a naturally occurring chemical compound, isolated from the bark of the cinchona tree, found mainly in the tropical forests of Peru, Bolivia, and Ecuador. The indigenous groups in these areas mixed the ground bark of cinchona trees with sugar water to offset the bitter taste, producing a beverage similar to today’s tonic water. QN was brought to Europe by early Spanish explorers and was initially used to treat malaria. Apart from quinine, the bark also contains quinidine, cinchonine, and cinchonidine, which most often are isolated as a mixture [1,2,3]. For this purpose, patients could take the appropriate amount of QN either by mouth, as pills in the form of salts or mineral acids, or by injection directly into their veins. QN pills were also used to treat lupus and arthritis, as well as to commit suicide. In the middle of the 20th century in many black-and-white Greek movies, the protagonist threatened to commit suicide by taking QN pills if the older brother did not give his approval to marry her lover. As of 2006, QN is no longer recommended by the World Health Organization (WHO) as a first-line treatment for malaria because there are other substances that are just as effective with fewer side effects, such as allergic reactions that can damage the heart and blood vessels [4]. QN continues to play a key role in the treatment of malaria in sub-Saharan Africa, in countries such as Cameroon and Uganda, and in other areas of high malaria incidence, and its use is not limited to WHO recommendations [5].

In more developed countries, the use of QN for uncomplicated cases of malaria decreased due to its toxicity and the introduction of newer and better-tolerated therapies. Currently, in the Western world, QN is used as a flavor component of carbonated beverages such as tonic water and bitter lemon drink mixers. It was discovered that mixing tonic water with gin made it much more enjoyable, producing the iconic gin and tonic cocktail [6]. The utility of QN as a flavoring agent in carbonated beverages is currently limited by the US Food and Drug Administration (FDA) to 83 mg L^−1^, with most commercial tonic waters containing QN in concentrations ranging from 25 to 60 mg L^−1^ [7]. Greek legislation has recognized a maximum concentration limit for QN of 100 mg L^−1^ in non-alcoholic beverages and 45 mg L^−1^ in beverages containing fruit juices [8].

QN, among its other uses, has been employed as a sensitizing agent due to its exceptional quantum fluorescence efficiency in acidic media [9]. QN has been utilized for the development of many chemiluminescence methods [10] with different oxidants such as cerium(IV) [11], diperiodatonickelate(IV) [12], potassium bromate [13], and permanganate [14].

In terms of analytical chemistry, there have been developed many analytical methods for the determination of QN in several samples, including biochemical and food samples. These methods are mainly instrumental, including batch spectrometry [15], atomic absorption spectroscopy (AAS) [16], capillary electrophoresis [17,18], electrochemistry [19,20,21], flow injection analysis (FIA) [22,23], chemiluminescence [24,25,26], liquid chromatography [27,28,29,30], mass spectrometry [31], and fluorescence [32,33].

Although these methods are accurate, reproducible, and provide low detection limits, they require trained personnel, expensive consumables and large volumes of solvents, and sophisticated instrumentation for the analytical determinations of such analytes. These drawbacks are major obstacles to the development of high-throughput methods when multiple samples must be analyzed in remote areas or in areas with reduced budgets. Motivated by these thoughts, we tried to develop a new fluorometric analytical paper-based method for the determination of QN in soft drink samples. Recently, by our research team, there has been a leap forward in the creation and development of low-cost and simple paper-based analytical devices, which can be applied with minimal resource and instrumentation requirements, to offer rapid and reliable results in the determination of various analytes [34,35,36,37]. Additionally, these paper-based sensors can very easily become portable and be placed at the point of need without losing the discrimination, accuracy, and repeatability of their measurements because the determination of the analyte can be carried out with a smartphone detector or flatbed scanner with fluorometric or colorimetric measurements [38,39,40]. Finally, a few outstanding research articles have been carried out for the fluorometric determination of QN using paper-based platforms with μPAD-compatible PEDD-based detectors [41] or 3D printable devices [42].

In an effort to further simplify the overall analytical process, we report herein a paper-based assay for the analytical determination of QN that depends on the fluorometric emission of its molecules in an acidic environment without any chemical reaction after the irradiation of the paper surface with a UV lamp at 365 nm. The paper platforms designed by our research group are cheap, easy, and fast to manufacture, and the analytical protocol used for such determination is easily feasible with minimal technical expertise and with no instrumentation. According to this, the analysis can be performed with the addition of the acid for the pH modification of the paper surface and the addition of the analyte (QN), the illumination of the device with a UV lamp, and the measurement of the fluorescence emitted on the sensing paper area, using a simple imaging device such as a smartphone. The method developed was tested for the determination of QN in tonic water, and the results were satisfactory in terms of sensitivity, recoveries, and reproducibility.

## 2. Materials and Methods

### 2.1. Reagents and Solutions

Nitric acid (HNO_3_) and quinine hemisulfate salt (QN) were purchased from Sigma (St. Louis, MO, USA). For the selectivity study, magnesium (Mg^2+^) standard solution (1000 mg L^−1^) in 0.5 mol L^−1^ HNO_3_, calcium (Ca^2+^) standard solution (1000 mg L^−1^) in 0.5 mol L^−1^ HNO_3_, sodium nitrate (NaNO_3_), potassium nitrate (KNO_3_), potassium chloride (KCl), sodium hydrogen carbonate (NaHCO_3_), and citric acid (C_6_H_8_O_7_) were provided by Merck (Darmstadt, Germany). Sugar was bought from local stores. For the study of the influence of ionic strength, sodium nitrate (NaNO_3_), sodium chloride (NaCl), and sodium bromide (NaBr) were provided by Panreac Quimica SA (Barecelona, Spain), Merck (Darmstadt, Germany), and May & Baker Co., Ltd. (Dagenham, UK), respectively. Analytical grade reagents were used for the experiments, while de-ionized water was used for their dissolution. The standard stock QN solution was 100 mg L^−1^ and produced daily in de-ionized water, and working QN solutions were prepared on a daily basis by diluting the stock solution at a final HNO_3_ concentration of 0.1 mol L^−1^. A stock solution of 2 mol L^−1^ HNO_3_ and working solutions of 0.1 mol L^−1^ were used for the pH adjustment on the surface of the paper devices. Cation and anion stock solutions for the selectivity study were 1000 mg L^−1^ for each ion studied. Citric acid stock solution was 0.262 g in 10 mL, and sugar stock solution was 2.0 g in 10 mL. Finally, sodium nitrate, sodium chloride, and sodium bromide stock solutions (2 mol L^−1^) and the following working solutions used for the salinity study were prepared by dissolving the appropriate amount in de-ionized water.

### 2.2. Apparatus

The fabrication of PADs was developed with a ColorQube 8580DN Xerox printer to accurately place solid wax on Whatman No. 1 chromatography paper in pre-crafted round patterns created on a white background. The pH values of the acidic solutions were adjusted with a pH meter (Orion). A UV illumination lamp (Vilber Lourmat) was used to irradiate the sensing areas of paper devices with UV light (365 nm), with an intensity of 15 W. The images of the devices were captured at specific time periods and at the same distance from the lamp using a mobile smartphone (Xiaomi Redmi Note 10, Xiaomi Corporation, Thessaloniki, Greece).

### 2.3. Manufacture of the Paper Devices

Hydrophilic sensing areas were designed using PowerPoint, and the patterns of the devices were printed on the paper using a solid ink printer. Subsequently, the paper was heated in a common laboratory oven for 2.0 min at 120 ± 5 °C to melt the wax and pierce the paper resulting in the creation of hydrophobic barriers on both sides of the paper. The paper-based devices produced from this procedure had a total diameter of 0.8 cm, an internal diameter that defined the hydrophilic sensing zone of 0.6 cm, and 0.2 cm wax barrier thickness. Chromatography paper (Whatman No. 1) was used for this procedure because of its homogenous configuration in comparison with other types of paper, such as filter paper, high thickness that prevented tearing or creating imperfections on the surface of the paper, relatively high mass per area (0.18 mm, 87 gm^−2^), and the absence of additives affecting the experimental process [43].

### 2.4. Experimental Process

**Figure 1:**
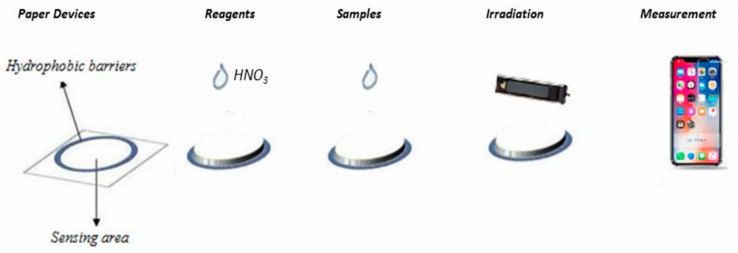
Experimental methodology of the proposed paper-based method.

The experimental procedure was easy to evolve, with almost no requirements for laboratory instrumentation. In brief, 0.1 mol L^−1^ of nitric acid (1 μL) and sample/standards (1 μL) were sequentially deposited at the paper surface. For the blank sample, de-ionized water was added to the device. Before any new solution addition, the paper device was left to dry at room temperature. Then, the sensor was irradiated under a UV lamp at 365 nm, and the fluorometric signal was captured using a smartphone. The photographs were saved as JPEG files (300 dpi analysis), and the fluorescence values were measured in RGB mode with Image J (Figure 1).

### 2.5. Real Samples

Soft drink samples and tonic water samples were purchased from local stores and were stored immediately in the refrigerator at 4 °C. The selectivity and sensitivity of the proposed method resulted in simple sample preparation, which included only the following easy and rapid steps: 2–4-fold dilution with de-ionized water depending on the levels of QN in the real samples, the addition of a few μL on the pH pretreated paper devices, and the determination of QN levels by the paper-based fluorometric method suggested.

### 2.6. Corroborative Batch Fluorescence Method

The batch fluorescence method was used as a corroborative method with a Shimadzu RF-5301pc spectrofluorophotometer and quartz cell of 1 cm path length. The wavelengths for excitation and emission of QN molecules were 360 and 460 nm, respectively. The calibration curve for the batch fluorescence determination was prepared by dissolving the appropriate amount of QN in de-ionized water. Working solutions were 2.0, 4.0, 6.0, 8.0, and 10 mg L^−1^. The calibration curve for the corroborative method was FL = 44.2C_QN_ + 16.4, with R^2^ = 0.997. Ten soft drinks were analyzed in triplicate with this method with only the appropriate dilutions (from ¼ to 1/8) to find the readings within the limits of the curve.

## 3. Results and Discussion

### 3.1. Optimization Parameters

The capability of the proposed paper-based procedure for the selective fluorometric determination of QN in tonic water samples was studied in detail, and all parameters that can affect the efficiency of this method were thoroughly studied and are subsequently presented.

#### 3.1.1. Effect of Acid Type

**Figure 2:**
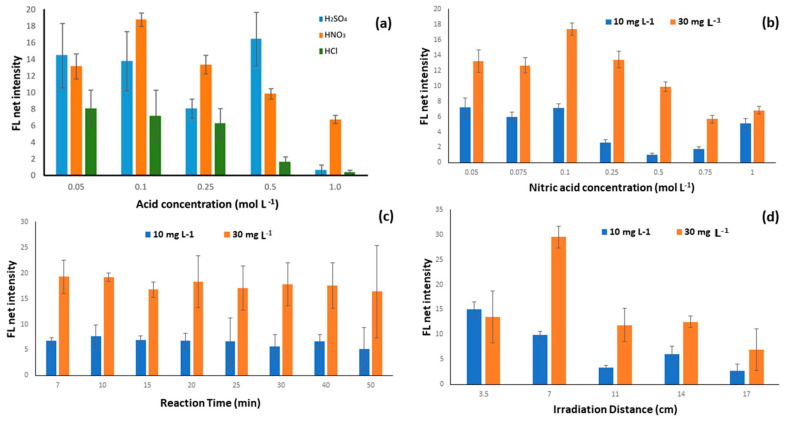
Influence of parameters on the fluorescence net signal for the proposed method.

QN is by far the most widely known fluorescent quantum performance standard used nowadays. The vast majority of the fluorometric analytical methods that have been developed to date in analytical protocols include the use of sulfuric acid, usually at a concentration of 0.05 mol L^−1^. However, recently, one research group looked for new ways to acidify QN samples. As a result, they proved that 0.05 mol L^−1^ sulfuric acid may lead to important errors caused by significant variations in the quantum yield at room temperature [44]. Thus, they proposed that perchloric acid should replace sulfuric acid during fluorometric QN determination [44]. Motivated by these research results, as well as the different substrate studied (paper instead of aqueous solutions), we decided that the first parameter we should study was the influence of different acids, for the pH adjustment, available in our laboratory. We tested the effect of three different acids at different concentrations. It is clear from Figure 2a that nitric acid was the best acid for setting the pH value on the surface of the paper devices in terms of signal intensity, as well as for the reproducibility of the measurements.

#### 3.1.2. Effect of Nitric Acid Concentration

After determining the type of acid that we would use in the analytical methodology, the influence of the HNO_3_ concentration in the evolution of the fluorescence reaction was studied in the range between 0 and 0.2 mol L^−1^ by the addition of 1 μL of the diluted solutions to the paper device. The nitric acid solutions studied were between 0.05 and 1.0 mol L^−1,^, and the corresponding pH values ranged from 1.30 to 0.00. Maximum fluorescence values were achieved for an HNO_3_ concentration of 0.1 mol L^−1^, and, thus, this concentration was adapted for all the experiments (Figure 2b).

#### 3.1.3. Effect of Reaction Time

Fluorescence is a rapid phenomenon even at room temperature, and its intensity can be measured immediately after the UV irradiation [40]. Nevertheless, due to the different matrices used during this study (paper), it was considered necessary to study the reaction time for the fluorometric quantitative determination of the QN molecules. Thus, the effect of the reaction time was studied in the range of 7 to 50 min, as depicted in Figure 2c. The mean fluorescence of both QN samples (10 and 30 mg L^−1^) was determined in triplicates, and no strong fluctuations were observed in the fluorescence of both the blank sample and the QN sample. The slight decrease in the fluorometric signal for the higher internal times was attributed to the quenching of the fluorescence after some time on the surface of the paper devices from the ambient laboratory light [40]. It is obvious from Figure 2c that the reception of the photograph can be taken at a reaction time of 7 to 20 min. We chose 10 min for time-consuming reasons, as well as to ensure that the paper devices are completely dry.

#### 3.1.4. Effect of Ionic Strength

A classic experiment to understand fluorescence by chemistry students is the addition of chloride anions to a QN solution in a high concentration in order to significantly quench the fluorescence emission. Many studies have been accomplished regarding the QN fluorescence quenching at different values of ionic strength [45,46]. Thus, three different sodium salts (NaNO_3_, NaCl, and NaBr) were tested to define if ionic strength affects the fluorescence signal of QN in both concentrations studied (10 and 30 mg L^−1^). The effect of ionic strength during the fluorescence emission was studied by adding different concentrations of NO_3_^−^, Cl^−^, and Br^−^ solutions to the paper surface just before the addition of the analyte. The reaction was not affected significantly by changes in ionic strength using NaNO_3_ from 0.025 mol L^−1^ up to 1.0 mol L^−1^, and in all cases, there was no more than approximately a 6% enhancement at the net fluorescence signal. On the contrary, chloride and bromide anions revealed a dramatic reduction in the analytical signal, as was expected, and even from the lowest concentration studied (0.025 mol L^−1^), a 40% and 57% decrease in the net fluorescence signal was determined for Cl^-^ and Br^-^ ions, respectively. However, the concentration of these anions in soft drinks such as tonic waters is negligible, and no quenching of fluorescence was anticipated. Thus, the net FL signal improvement of sodium nitrate addition was not sufficient to add an extra step in the method, and no salt addition was chosen throughout the experiments.

#### 3.1.5. Irradiation Distance

In acidic media, QN has two analytically useful excitation wavelengths at 250 and 350 nm. However, the maximum emission wavelength is always 450 nm, regardless of the excitation wavelength studied. So, the choice of the excitation wavelength and the distance of the paper devices from the UV source are critical for the optimization of this method. Consequently, two different lamps with excitation wavelengths at 254 and 365 nm, respectively, were studied. The net signal (signal of the sample minus the signal of the blank) measured at 254 nm was 10 to 12% lower than that at 365 nm, and, thus, all experiments were conducted with the excitation of the paper-based devices at 365 nm.

The distance from the UV light source is also an important parameter as it significantly affects the amount of fluorescence to be measured. Thus, a variety of different distances from the light source were studied from 3.5 to 17.0 cm, and that of 7.0 cm was found to be the optimum one for this method (Figure 2d). Smaller distances than 3.5 cm were not sufficient because there was not enough available space between the smartphone camera and the paper device for a clear and stable photo to be taken, while for higher distances the intensity of the radiation reaching the paper was significantly reduced with a consequent decrease in fluorescence measured, as shown in Figure 2d.

#### 3.1.6. Effect of the Image Capture Procedure

When performing fluorescence measurements with a smartphone, the irradiation lamp position as well as the smartphone position must be adapted so that the ambient or scattered light is limited as much as possible [38]. In order to avoid the influence of diffused light and the change in the angle of taking the photograph during the determination of QN, many variations of the procedure were tested. Initially, the lamp was placed parallel to the lab bench at various heights from the paper apparatus, with the simultaneous absence of sunlight but with the lab lights on. The lamp was then placed back parallel to the lab bench and surrounded by a handmade dark-colored box, with a hole in the front capable of holding a smartphone. In both of these cases, the results were poor as the repeatability of the method was significantly affected by diffused light and intense glare from the high intensity of the irradiation lamp. Then, the excitation lamp, using a metal adapter, was set at an angle of 45 degrees and moved 5–20 cm away from the paper device while the dark box surrounded it and completely repelled the diffused light. The mobile phone was placed at various shooting angles (from perpendicular to almost parallel) to the paper devices in special holes created on the box. The best result was obtained for a fixed shooting angle perpendicular to the surface of the paper with the lamp at a distance of 10 cm and at an angle of 45 degrees, and this methodology was consistently used for the continuation of the experiments.

#### 3.1.7. Effect of the Detection Zone Size

The paper-based analytical devices used during this research were the simplest ones, made with a circular hydrophobic wax barrier (Figure 1). Τhis circular shape can be configured in various diameters and sizes of the detection area, and the question that arises is whether these different sizes can affect the amount of fluorescence. So, three different sizes of the detection zone were studied. Specifically, a large, a medium, and a small configuration of these circular hydrophobic arrangements with total diameters of 0.9, 0.8, and 0.7 cm, respectively, were studied. A standard QN solution of 30 mg L^−1^ and a 0.1 mol L^−1^ nitric acid solution were prepared for this analysis, and after the deposition of each solution on the paper surface, the samples were allowed at room temperature to dry for 10 min. In the largest device (inner diameter of 0.5 cm and total diameter of 0.9 cm), it was observed that the volume of 1 μL was not enough to spread over the entire surface of the paper, and, thus, the measurements had poor repeatability. Additionally, the small device with an inner diameter of 0.3 cm and a total diameter of 0.7 cm performed with a fluorescence intensity value of 13.1 ± 0.5, while the middle device with a 0.4 cm inner diameter and a 0.8 cm total diameter revealed a fluorescence intensity value of 15.8 ± 0.8. Therefore, the middle size of the paper devices was chosen for the even distribution of the reagents, as well as the higher fluorescence net signal.

### 3.2. Method Validation

The proposed paper-based method was validated in terms of accuracy, linearity, limits of detection (LOD) and quantification (LOQ), precision, selectivity, and stability of the PADs.

#### 3.2.1. Linearity, Precision, and Limits of Detection (LOD) and Quantification (LOQ)

**Figure 3:**
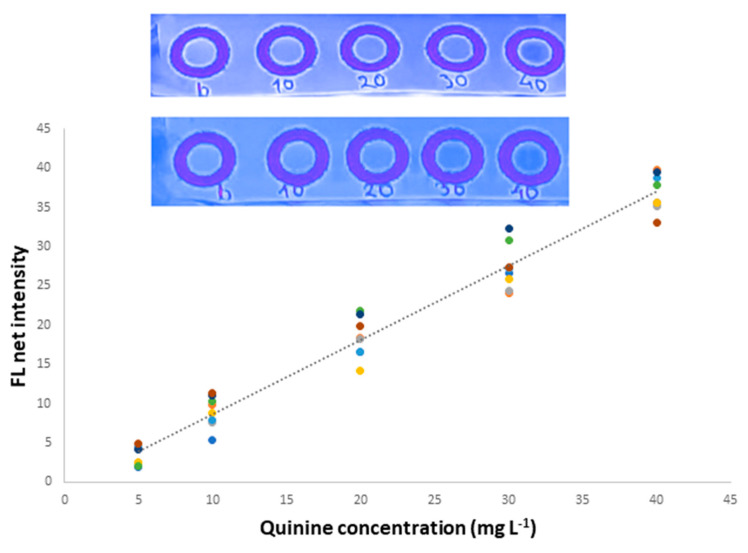
Cumulative calibration curve for the proposed method. Integrated photo: Fluorescence signal obtained for the plot of the calibration curve.

The developed method showed the adequate linearity of QN concentrations in the range between 5 and 40 mg L^−1^ (Figure 3). For the validation of the method, the regression equation was obtained in a “cumulative” manner by incorporating the results from 40 standard solutions measured in different working days (*n* = 8). By this, the calibration curve is more vicarious, including possible day-to-day alterations, and the subsequent regression equation was obtained:

FL = 0.960 (±0.044) [QN] − 1.49 (±1.04), R^2^ = 0.969

where FL is the fluorescence intensity measured by the paper-based method.

The within-day precision was validated for the 10.0 and 30.0 mg L^−1^ levels by repetitive measurements of different paper sensing areas (*n* = 5), and the intra-day relative standard deviation (RSD) was 3.1% and 7.3% for these concentration levels, respectively. The inter-day precision was 5.5% and 8.8% for the same concentration levels, respectively. Finally, the LOD and LOQ values were calculated as LOD = 3.3 × SDb/s and LOQ = 10 × SDb/s, where SDb is the standard deviation of the intercept, and s is the slope of the respective regression lines. The calculated LOD/LOQ for the analysis of QN were 3.6 and 10.8 mg L^−1,^, respectively.

#### 3.2.2. Interference Study-Selectivity

**Figure 4:**
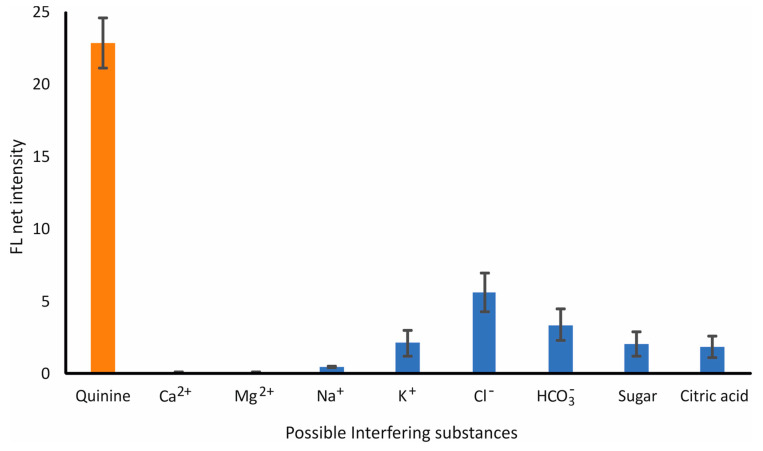
Selectivity of QN determination under the optimum experimental conditions (QN: 30 mg L^−1^; HNO_3_: 0.1 M; irradiation distance: 7 cm; reaction time: 10 min). Error bars are the standard deviation for *n* = 3.

The interference study of the developed sensor was validated against common cations, anions, and molecules that were expected to be present in the tonic water samples. Based on this, Ca^2+^, Mg^2+^, Na^+^, K^+^, Cl^−^, HCO_3_^−^, sugar, and citric acid were analyzed at 75, 25, 25, 10, 20, 400, 2 × 10^5^, and 26.2 × 10^3^ mg L^−1^, respectively, whereas QN was analyzed at 30 mg L^−1^. The experimental results are shown in Figure 4 and verified the selectivity of the procedure, considering the expected levels and ratios of QN in the tonic water samples with no significant interference observed.

#### 3.2.3. Time and Storage Stability of the Devices

The portability of paper-based devices is one of the most important advantages of such methods for their applicability in field analysis. To evaluate the portability and the stability of these devices, we studied the μPADs by adding the nitric acid solutions on each device and storing them in airtight bags protected from light at room temperature (25 °C), refrigerator (+4 °C) and freeze (−18 °C). The stability was examined at two levels of QN: 10 and 30 mg L^−1^ after 2 days, 4 days, and 6 days of storage. The experimental results as % recoveries of QN are summarized in Table 1. The estimated percent recoveries for the μPADs are stable and usable even after 6 days if kept at −18 °C and protected from the light; otherwise, the stability of these devices is rapidly decreased after 4 days at room temperature or at +4 °C.

#### 3.2.4. Real Samples Applicability and Comparison with a Fluorescence Method

The applicability of the developed method was evaluated by the measurement of ten tonic water samples. As anticipated, the tonic water samples had QN values higher than the calibration curve limits; thus, the samples were diluted two to four times. The results are presented in Table 2. The evaluation of the accuracy of the developed method included an analysis of the same tonic water samples by a standard batch fluorescence method (see Section 2.6. for experimental details). The results and the calculated relative errors (%) are summarized in Table 2. Only the second sample studied showed a fairly significant relative error (−19.2%), which is attributed to its low QN content below the detection limit of the method. Nevertheless, the trend in this sample is also remarkably close to the actual value measured by the specified fluorometric method. The rest of the calculated relative errors were between −7.4% and +14.4% for the real samples, indicating that the average accuracies are satisfactory, and the method developed for QN determination in the tonic water samples was in good agreement with the reference method.

## 4. Conclusions

A fast, equipment free, and reliable paper-based method for the selective determination of QN in soft drink samples has been developed and validated. The µPADs used during this study were easily fabricated at a minimal cost. The developed paper-based sensor utilized only two readily available reagents and is desirable for real-time applications. The developed analytical method was based on the fluorescence generated at strongly acidic conditions, and the fluorescence emission was recorded by a simple camera of a smartphone. The irradiation was achieved by a commercially available UV lamp at 365 nm, and the measurement lasted for a few seconds. Additionally, the analytical methodology developed is robust, can analyze many samples in a short time period, and permits the analysis of QN at low microgram levels (LOD 3.6 mg L^−1^). The levels of QN in the analyzed samples were within the expected limits allowed by the World Health Organization and were also confirmed by a confirmatory batch fluorescence method. The levels of QN in the analyzed real samples were within acceptable recovery limits from 92.5 to 114.3%, as confirmed by the corroborative fluorescence method.

## Figures and Tables

**Table 1:** Stability of the μ-PADS (HNO_3_) under different storage conditions.

	Time (days)		
	2	4	6
Temperature (°C)		% Recovery	
25	103.5 ± 7.8	88.0 ± 5.2	37.6 ± 12.9
4	106.0 ± 2.4	95.9 ± 10.5	68.1 ± 8.7
−18	96.8 ± 1.5	93.5 ± 4.8	89.5 ± 5.3

**Table 2:** Paper-based method vs. the batch fluorescence method.

Sample	QN Found (mg L^−1^) ^a^		
	This Method	Fluorescence Batch Method	(%) Relative Error
1	38.7 ± 1.3	37.0	+4.6
2	2.1 ± 0.2	2.6	−19.2
3	55.5 ± 1.6	56.8	+2.3
4	48.0 ± 6.1	44.6	+7.6
5	70.5 ± 1.9	75.1	−6.1
6	56.0 ± 2.0	57.5	+2.6
7	40.3 ± 1.1	41.1	−1.9
8	50.1 ± 1.2	43.8	+14.4
9	44.5 ± 1.3	42.5	+4.7
10	41.1 ± 2.8	44.4	−7.4

^a^ Mean of three measurements.

## Data Availability

Not applicable.
